# Using the intervention mapping for adaption framework to adapt an evidence-based sexual health intervention for youth affected by trauma

**DOI:** 10.1186/s12889-023-15984-2

**Published:** 2023-06-01

**Authors:** Olivia N. Kachingwe, Quiana Lewis, Asari Offiong, Bianca D. Smith, Ashleigh LoVette, Terrinieka W. Powell

**Affiliations:** 1 Department of Population, Family and Reproductive Health, Johns Hopkins Bloomberg School of Public Health, Baltimore, MD United States of America; 2 Child Trends, Washington, DC United States of America; 3 College of Public Health, The Ohio State University, Columbus, OH United States of America

**Keywords:** Sexual health, Black youth, Intervention mapping-adapt, Adaption, FOY + ImPACT

## Abstract

**Background:**

Children exposed to household challenges (i.e., parental substance use, incarceration, and mental illness) are among the groups most vulnerable to sexual risk-taking in adolescence. These behaviors have been associated with a range of negative outcomes later in life, including substance abuse, low educational attainment, and incarceration. Adapting an evidence-based intervention (EBI) to be suitable for this population is one strategy to address the needs of this group.

**Methods:**

In this study, we describe the use of the Intervention Mapping for Adaption (IM-Adapt) framework to adapt an evidence-based, sexual health intervention (*Focus on Youth with Informed Children and Parents*). We describe the actions taken at each step of the IM-Adapt process which are to assess needs, search for EBIs, assess fit and plan adaptions, make adaptions, plan for implementation and plan for evaluation.

**Results:**

Key changes of the adapted intervention include the incorporation of trauma-informed principles and gender inclusive language, standardization of the session length, and modernization of the content to be more appropriate for our priority population.

**Conclusions:**

The adapted intervention shows promise toward meeting the behavioral health needs of Black youth exposed to household challenges. Our process and approach can serve as a model for researchers and practitioners aiming to extend the reach of EBIs.

## Introduction

The association between exposure to adverse childhood experiences (ACEs), chronic diseases, mental illnesses, and negative health behaviors has been well established [1]. In the United States, more than two-thirds of all children experience at least one ACE [2, 3]. Household challenges, which are a subset of ACEs, include experiences of parental substance use, parental incarceration, and parental mental illness [2]. Approximately, one in eight children are affected by parental substance use disorders, parental incarceration, or parental mental illness [4–6]. Black youth experience a higher burden of household challenges [7]. Exposure to household challenges is associated with a range of negative health outcomes later in life including substance use dependency, low educational attainment and incarceration [8–10].

Exposure to household challenges may make it difficult for youth to make healthy decisions and increases their likelihood of engaging in sexual risk behaviors such as unprotected sex and experiencing an unintended pregnancy [11–13]. Black teens have significantly higher rates of sexually transmitted infections (STIs), including chlamydia, gonorrhea, and syphilis when compared to the national average [14]. The birth rate among Black teens aged 15 to 19 is more than 1.5 times higher than the national average (24.4 vs. 15.4 per 1,000 females, respectively) [15]. Black youth are more likely to report having used alcohol or drugs prior to their last sexual intercourse when compared to their White peers, which is positively associated with contraception non-use (a risk factor for unintended pregnancy and contracting HIV/STIs) [11, 16, 17]. Taken together, these findings suggest that tailored interventions that consider the social context and lived experiences of youth are needed to improve sexual health outcomes for Black youth exposed to household challenges [18–20].

*Focus on Youth with Informed Parents and Children Together* (FOY + ImPACT) is an evidence-based skill-building intervention that is aimed at preventing substance use and sexual risk-taking among high-risk African American youth. It has two components: Focus on Youth (FOY) and ImPACT. The FOY component is an 8-session intervention for youth only that emphasizes core components of other evidence-based substance use prevention programs, such as decision making, goal setting, communication, and negotiation. The ImPACT component is a single-session intervention for youth and caregivers, where families receive information about sexual health topics and practice communication skills.

Despite its strong core components and proven effectiveness, FOY + ImPACT does not reflect the experiences of today’s youth, nor does it incorporate trauma-informed principles [21] necessary for effectively engaging youth exposed to household challenges. Adaptions are a common, affordable and time efficient approach to extending the reach and impact of evidence-based interventions (EBIs). In this paper, we describe how we used the Intervention Mapping for Adaption framework to adapt the FOY + ImPACT intervention to be suitable for Black youth exposed to household challenges.

## Methods

For the purposes of this paper, adaption was defined as the process of making changes to a program to make it more suitable for a particular population and new organization [22]. Intervention Mapping for Adaption  (IM-Adapt) is a six-step systematic approach to guide decision making in each phase of adapting an EBI. The six steps are as follows: assess needs, search for EBIs, assess fit and plan adaptions, make adaptions, plan for implementation and plan for evaluation [23]. Table 1 provides a short description of each step. Below we describe our process using IM-Adapt to adapt FOY + ImPACT to be suitable for Black youth exposed to household challenges.

**Table 1:** Steps to Intervention Mapping for Adapt EBIs

Intervention Mapping Step	Description
Step 1: Assess needs	Describe the problem and organizational capacity. Develop a logic model and program goals.
Step 2: Search for EBIs	Search for evidence-based interventions that address the health behavior among the priority population
Step 3: Assess fit and plan adaptions	Assess intervention behavioral and environmental fit, determinants of change, delivery, design, and cultural appropriateness.
Step 4: Make adaptions	Adapt intervention materials and assessment tools
Step 5: Plan for implementation	Identify implementation sites, develop facilitation scope, train facilitators, and plan implementation logistics
Step 6: Plan for evaluation	Develop evaluation tools and evaluation design including the data collection protocol, analysis and reporting

### Step one: assess needs

Thirty in-depth interviews were conducted with key informants including parents with a history of drug use (n = 11), young adults exposed to household challenges (n = 14), and service providers who work with families exposed to household challenges (n = 5). We obtained information across themes related to personal history, family relationships, health, and program recommendations. Data were analyzed inductively using a content analytic approach [24] and Atlas.ti (version 8). Participants described a lack of parental support and frequent turnover in guardianship care. Not having a strong connection to one’s parent made it challenging to trust other adults, often leading to expected disappointment. Participants desired consistent parental involvement and support from other adults in their lives to help ensure their emotional and physical well-being [25]. As a result, we chose to center the program on trauma-informed principles. These principles include safety, trustworthiness and transparency, peer support, collaboration and mutuality, empowerment and choice, and responsiveness to culture, historical and gender issues [21].

### Step two: search for EBIs & assess fit

To obtain a broad view of effective interventions focused on sexual health, we reviewed the *Compendium of Evidence-Based Interventions and Best Practices for HIV Prevention* [26] and the *Teen Pregnancy Prevention Evidence Review* [27]. These websites provided descriptions of general approaches, topics discussed, priority populations, and a summary of findings. To obtain more specific information about potential interventions, we reviewed articles detailing the methods and evaluation results of each intervention.

FOY + ImPACT was selected for four reasons. First, FOY + ImPACT is one of few interventions to report significant reductions in substance use and sexual risk-taking among adolescents. Intervention participants were less likely than control participants to report sexual intercourse and unprotected sex at 6-month follow-up, cigarette smoking at 6- and 24-month follow-up, alcohol use at 6- and 12-month follow-up, marijuana use at the 12-month follow-up, and pregnancy/impregnation at 24-month follow-up [28–30]. Second, it was developed and tested with poor, high-need African American youth living in public housing developments in Baltimore which best aligns with our priority population. Our priority population was Black youth ages 13 to 16 exposed to household challenges. Third, the developers trained local facilitators to recruit participants, collect data, and successfully implement the intervention [31]. We also trained lay people from community organizations to recruit participants, and aid in data collection. Fourth, the intervention aligns well with the capacity of community settings due to its flexibility around session offerings and inclusiveness of all caregivers.

### Steps three and four: plan and make adaptions

The adaption of FOY + ImPACT occurred in three stages. The research team first partnered with a local videographer to develop an updated version of the caregiver video used during the ImPACT session. Second, we identified additional content by reviewing the literature, findings from the needs assessment, and materials from prior adaptions of FOY + ImPACT such as Respecting the Circle of Life [32]. To ensure intervention fidelity, our team retained the core elements of the original intervention, such as its delivery in a community setting that is welcoming, safe and easily accessible. Third, the adapted intervention was reviewed by two external researchers who have extensive expertise in developing interventions for youth, one of which helped develop the original FOY + ImPACT. This reviewer ensured that modifications of key characteristics in the adapted intervention do not compete with or contradict core elements, theory, and internal logic of the original intervention [33].

### Steps five and six: plan for implementation and evaluation

The implementation team consisted of trained research assistants from various racial backgrounds, all of whom identified as female ages 22 to 31. The desired implementation outcome was the delivery of the intervention to 60 youth and the delivery of the control condition to 60 youth, for a total of 120 enrolled youth. Youth in the control condition received group sessions focused on literacy and career opportunities. The original implementation tools and manual were helpful but not sufficient for our context. Therefore, the research team developed training sessions to encourage self-efficacy, outcome expectations, and facilitation skills of the implementers. The skills included opening, moving, and closing conversations, establishing rapport, conducting active listening, and addressing barriers. In the training sessions, we explained the theory behind the program, and spent a substantial amount of the time conducting role play practice with feedback.

Our evaluation plan sought to determine the initial efficacy of the adapted FOY + ImPACT on sexual health outcomes for youth exposed to household challenges. We used the following question to guide our effectiveness evaluation: Did youth who receive the adapted FOY + ImPACT report lower rates of sexual risk taking (i.e., initiation of sex, frequency of unprotected sex, and frequency of substance use during sex) when compared to youth who received the control condition? The evaluation for the adapted FOY + ImPACT is currently in progress.

## Results

### Adapted FOY + ImPACT

The current manuscript focuses on the adaption of FOY + ImPACT (IM-Adapt Step Four). To ensure that the curriculum was relevant to the experiences of our priority population, we adapted FOY + ImPACT in seven ways. First, we re-filmed the video used during the ImPACT session to ensure that the video accurately reflected the lived experiences of families exposed to household challenges. Second, we standardized the length of each session to be 100 min to accommodate our partner organizations’ schedules more easily. Third, we added gender-inclusive language and examples of queer relationships so that the adapted FOY + ImPACT is representative of diverse lived experiences and identities. Among youth in 9th to 12th grade, 15.6% do not identify as straight or heterosexual [34]. The introduction of queer-centered content was supported by prompts and thoughtful questions throughout the curriculum, encouraging participants to reflect on how one’s sexual orientation, gender identity and community context may intersect and collectively impact decision-making.

Fourth, given the priority population, trauma-informed principles were applied to the adaption process and curricula changes. For example, we added notes to facilitators throughout the curriculum encouraging them to help youth (1) identify safe people and places and (2) strategize ways to collaborate effectively with their identified support network. Further, we removed stigmatized language and updated the language throughout the curriculum from “parents/guardians” to “caregivers and other trusted adults.” This allows youth who lack consistent parental involvement to participate in ImPACT with another trusted adult that does not possess legal guardianship (e.g., a church pastor or alternate family member). To further ensure that youth feel safe and empowered throughout all sessions, the updated curriculum stipulates that qualified intervention facilitators must be knowledgeable of local resources, sexual health-related state policies and trained in trauma-informed principles. Finally, to account for the unique experiences of our priority population and to increase intervention acceptability, content geared towards dealing with household challenges (i.e., network building, coping strategies and linkages to community resources) and substance use, addiction and recovery were also added to the curriculum. For example, in one roleplay scenario, the effects of substance use on poor decision-making were explicitly highlighted.

Fifth, the original FOY + ImPACT curriculum uses the Stop, Options, Decide, and Act (SODA) decision-making model. However, previous studies suggest that youth experiencing household challenges are often forced to make decisions when no ideal options are available [35]. Therefore, the acronym, BEST, was created to encourage youth to make the “best” decisions for themselves, despite their circumstances. Our adapted decision-making model instructed youth to Breathe, Educate yourself, Select the best option for you, and Take action (BEST). Developed through a trauma-informed lens, this acronym highlights the strengths and assets youth have with the word “best” being affirming and positive, and easy to remember.

Sixth, we introduced the use of social media during one of the roleplay scenarios presented during the ImPACT session. The use of social media is highest among young people, with 84% of those ages 18 to 29 reporting having ever used any social media site [36]. Many youth and young adults currently use social media to communicate with their intimate partners or to seek new relationships. However, social media can have both positive and negative effects on youth’s relationships, so it is important that youth have the tools needed to safely navigate their on-line interactions [37–40]. Thus, it was a priority to ensure that youth ages 13 to 16 and their trusted adults were familiar with the potential benefits and harm associated with social media use.

Finally, major revisions were also made to the main storyline presented during the FOY discussions and activities. Specifically, we renamed the central “family tree” activity to be “circle of influencers” to reflect terminology well known to youth who use social media. An influencer is a social media user with a large following who can affect other users’ behavior by leveraging their popularity and trustworthiness. We also added characters to the circle of influencers who represented different spheres of influence (i.e., friends, teachers, community members, etc.), grounded in Bronfenbrenner’s social-ecological model [41]. Finally, we provided a detailed description of each characters’ lived experiences and social context which allows for richer, more directed dialogue. Table 2 provides a summary of the adaptions made.

**Table 2:** FOY + ImPACT Adaptions

	Original FOY + ImPACT Titles and Objectives	Adapted FOY + ImPACT Titles and Objectives	Key Changes
**Session 1**	We’re All in This Together Establish a cohesive group, by setting group agreements and participating in a group cohesion activity. Begin learning skills for decision making	Breathe: We’re All in This Together Establish group cohesion and begin to learn skills for healthy decision making.	Replaced family tree with Circle of Influencers Replaced S.O.D.A. with B.E.S.T. model
**Session 2**	Risks and Values Examine risk behaviors and why young people may feel invincible or invulnerable to understand how this can place them at risk for HIV/STD or unplanned pregnancy. Identify personal values through discussion, ranking and voting activities	Breathe: Knowing My Standards Learn facts about sex, pregnancy, STIs and HIV, and use the information to establish personal standards for one’s sexual health behavior.	Added discussion of community level influences on decision-making
**Session 3**	Educate Yourself: Obtaining Information Learn ways to obtain information in order to make good decisions by applying the decision-making model and researching answers to questions	Educate Yourself: Sex Ed 101 Learn information about sexual behavior risks, condoms and contraceptives that enable one to maintain safer sex standards.	Revised session to focus on sexual health knowledge and safe sex skills building
**Session 4**	Educate Yourself: Examining Consequences Learn to weigh the positive and negative consequences of options as they make decisions.	Educate Yourself: Finding Information Learn and practice ways to obtain sexual health information.	Incorporated information and activities for finding reliable information from trusted people and resources.
**Session 5**	Build Skills: Communication Learn communication and negotiation skills to assist in carrying out responsible decisions.	Select & Take Action: Communicating My Values & Consent Learn communication and negotiation skills to assist in carrying out responsible decisions.	Integrated conversations about consent and sexual violence across multiple activities
**Session 6**	Sexual Health and Showing you Care Without Having Sex Use roleplays to explore various ways to show they care without having sex and will learn information about sexual health.	Select & Take Action: More Than Sex Explore ways to demonstrate care and establish intimacy without having sex.	Removed HIV transmission game and added activities to help participants think of non-sexual ways to show affection.
**Session 7**	Attitudes and Skills for Sexual Health Learn attitudes and skills that support sexual health through listening to a speaker, completing a goal-setting activity and roleplaying refusal and negotiation skills.	B.E.S.T. Decision for you: Power to Decide Observe the consequences of decision-making through listening to a guest speaker. Set personal goals for the future.	Reframed goal setting activity to specifically focus on STI, HIV, unintended pregnancy and substance use. Used the BEST model to set goals
**Session 8**	Review and Community Project Build self-efficacy about HIV/STD prevention through analyzing their concerns and how they can take responsibility, testing their HIV knowledge, affirming each other and planning community projects.	B.E.S.T. Decision for you: Review and Community Project Build pregnancy and HIV/STI prevention self-efficacy. Review and teach-back key lessons learned.	Shortened session by focusing on reviewing content from the previous session and integrating the information learned by tasking participants to develop a HIV/STI or pregnancy prevention social media campaign.

## Discussion

We described use of the IM-Adapt framework to adapt an evidence-based sexual health intervention for a new population (i.e., Black youth exposed to household challenges). The IM-Adapt was a useful tool to guide our systematic adaptation. Interventions designed to prevent multiple risk behaviors in adolescents exposed to household challenges have had little success [42–44]. The adapted version of FOY + ImPACT integrated trauma-informed principles and emphasized the importance of trusted adults and community resources in the lives of youth exposed to household challenges. This adaption was also responsive to youths’ current social environment, use of social media, and diverse intimate relationships. Taken together, there is promise that the adapted FOY + ImPACT intervention is useful for Black youth exposed to household challenges.

The adapted version of FOY + ImPACT used trauma-informed principles to guide decisions around content, facilitator notes, and required facilitator training. Using a trauma-informed lens to guide public health intervention development and adaption is vital as knowledge is often necessary but not sufficient for effective behavior change. A history of trauma impacts how youth respond in situations related to sex, can limit one’s ability to cope, impede one’s decision-making process, and negatively impact cognitive, social and emotional development [21]. Sexual health interventions guided by trauma-informed principles and content have proven effective at increasing birth control use among youth experiencing homelessness, sexual health knowledge and attitudes among adolescent boys, and sexual health knowledge, communication and refusal skills among teen peer-educators [45–47]. Although the strength of using trauma-informed principles to inform behavioral health interventions has been established, few sexual health interventions address trauma in the programmatic content [48]. Notably, trauma-informed sex education is greatly supported by parents, especially parents who acknowledge that youth are exposed to high levels of trauma [49]. To this end, using a trauma-informed lens is a huge strength of the adapted FOY + ImPACT curriculum that we anticipate will increase its short and long-term impact on youth behavior change.

The success of youth-focused interventions in part hinges on their responsiveness to youth’s needs and adaptability to unforeseen implementation challenges. For this reason, the research team ensured that the adapted intervention would still be appropriate for various community settings. The standard length of each session also allowed for implementation flexibility, and for community sites to have more input in when and where sessions occurred. When delivering community-based interventions remaining flexible was recommended by previous interventionists who have adapted pre-existing programs [50]. The adapted FOY + ImPACT also incorporated language inclusive of youth’s diverse lived experiences (e.g., trusted adults vs. parents/guardians and the representation of non-gender conforming or non-binary identities and queer relationships). Using language that resonates with participants is an example of cultural sensitivity (more specifically surface structure) which has been shown to improve program comprehension and receptivity [51].

Our current evaluation of the adapted FOY + ImPACT considers the social-political environment where youth and their trusted adults are living. Shifts in federal and national policies resulting from the overturning of the 1973 Roe vs. Wade U.S. Supreme Court decision directly impact intervention relevancy and may warrant additional programmatic content to dispel myths or misinformation. Similarly, the educational environment (i.e., local teachers’ instructional pedagogies, instructional style, and educational tools) must also be considered and emulated, when possible, to circumvent a participant learning curve and aid program acceptability. Inviting local educators or community instructors and community-involved leaders to review the curriculum prior to its implantation is advised. Lastly, the utility of youth-serving community organizations as possible delivery sites must be assessed on an individual basis. Beyond programmatic interest and participant reach, organizations expressed varying degrees of staffing availability and physical space constraints. This raised challenges when identifying partnering organizations and required flexibility from the program implementors.

### Strengths and limitations

There are several limitations to consider in the FOY + ImPACT intervention adaption. First, the key-informants interviewed for the needs assessment lived in one Mid-Atlantic city in the United States. The recommendations provided might be unique to youth exposed to household challenges who live in this geographic area. Notably, the needs assessment was conducted prior to the COVID-19 pandemic, the adaption was conducted during the pandemic and the implementation and evaluation are in progress (post-pandemic). As a result, the experiences and needs of youth exposed to household challenges may have changed. Thus, our version of FOY + IMPACT may not reflect the additional supports needed. Further, because of COVID-19, appropriate venue-based groups might have changed, impacting where youth are gathering and building social networks. Potential gaps in the curriculum’s content and unforeseen implementation needs will be revealed through program delivery.

Despite these limitations, there are several strengths of note. First, this study provided a process through which evidence-based sexual health interventions delivered to youth exposed to household challenges can be adapted. Second, this paper highlighted the importance of exploring how data collected from families exposed to household challenges and service providers can be translated to intervention delivery. Third, adaptions made to the FOY + ImPACT intervention aligned well with current trends in sexual health education. Specifically, it included trauma-informed principles, libraries as a trusted health-resource, inclusion of caregivers and providers in the ImPACT video, an introduction of social media relative to sexual health communication, and content inclusive of diverse, queer relationships. Fourth, we demonstrated ways to make interventions more accessible by standardizing the session length and involving community partners.

## Data Availability

All data generated or analyzed during this study are included in this published article.

## Abbreviations

ACEs: Adverse childhood experiences
EBIs: Evidence-based Interventions
FOY + ImPACT: Focus on Youth with Informed Parents and Children Together
IM-Adapt: Intervention Mapping for Adaptation
STIs: Sexually transmitted infections
